# Exosomal Carboxypeptidase E (CPE) and CPE-shRNA-Loaded Exosomes Regulate Metastatic Phenotype of Tumor Cells

**DOI:** 10.3390/ijms23063113

**Published:** 2022-03-14

**Authors:** Sangeetha Hareendran, Bassam Albraidy, Xuyu Yang, Aiyi Liu, Anne Breggia, Clark C. Chen, Y. Peng Loh

**Affiliations:** 1 Section on Cellular Neurobiology, *Eunice Kennedy Shriver* National Institute of Child Health and Human Development, National Institutes of Health, Bethesda, MD 20892, USA; sangeetha.hareendran@nih.gov (S.H.); balbr025@uottawa.ca (B.A.); xuyu.yang@nih.gov (X.Y.); 2 Biostatistics & Bioinformatics Branch, *Eunice Kennedy Shriver* National Institute of Child Health and Human Development, National Institutes of Health, Bethesda, MD 20892, USA; liua@mail.nih.gov; 3 Maine Medical Center BioBank, Portland, ME 04074, USA; bregga@mmc.org; 4 Department of Neurosurgery, University of Minnesota Medical School, Minneapolis, MN 55455, USA; ccchen@umn.edu

**Keywords:** cancer proliferation, hepatocellular carcinoma, metastasis, engineered exosomes, diagnostic biomarker, cancer therapy

## Abstract

Background: Exosomes promote tumor growth and metastasis through intercellular communication, although the mechanism remains elusive. Carboxypeptidase E (CPE) supports the progression of different cancers, including hepatocellular carcinoma (HCC). Here, we investigated whether CPE is the bioactive cargo within exosomes, and whether it contributes to tumorigenesis, using HCC cell lines as a cancer model. Methods: Exosomes were isolated from supernatant media of cancer cells, or human sera. mRNA and protein expression were analyzed using PCR and Western blot. Low-metastatic HCC97L cells were incubated with exosomes derived from high-metastatic HCC97H cells. In other experiments, HCC97H cells were incubated with CPE-shRNA-loaded exosomes. Cell proliferation and invasion were assessed using MTT, colony formation, and matrigel invasion assays. Results: Exosomes released from cancer cells contain *CPE* mRNA and protein. *CPE* mRNA levels are enriched in exosomes secreted from high- versus low-metastastic cells, across various cancer types. In a pilot study, significantly higher *CPE* copy numbers were found in serum exosomes from cancer patients compared to healthy subjects. HCC97L cells, treated with exosomes derived from HCC97H cells, displayed enhanced proliferation and invasion; however, exosomes from HCC97H cells pre-treated with CPE-shRNA failed to promote proliferation. When HEK293T exosomes loaded with CPE-shRNA were incubated with HCC97H cells, the expression of CPE, Cyclin D1, a cell-cycle regulatory protein and *c-myc*, a proto-oncogene, were suppressed, resulting in the diminished proliferation of HCC97H cells. Conclusions: We identified CPE as an exosomal bioactive molecule driving the growth and invasion of low-metastatic HCC cells. CPE-shRNA loaded exosomes can inhibit malignant tumor cell proliferation via Cyclin D1 and c-MYC suppression. Thus, CPE is a key player in the exosome transmission of tumorigenesis, and the exosome-based delivery of CPE-shRNA offers a potential treatment for tumor progression. Notably, measuring CPE transcript levels in serum exosomes from cancer patients could have potential liquid biopsy applications.

## 1. Introduction

Exosomes are nano-sized extracellular vesicles (30–140 nm in diameter), which facilitate critical intercellular communication by way of transferring bioactive molecules. While exosomes are secreted by most cells, it is important to note that exosomes derived from tumor cells have a distinctly different composition to those released from healthy cells [1]. Tumor-derived exosomes are known to promote the tumorigenesis, metastasis, and modulation of the tumor microenvironment [2,3,4]. Recent reports have shown that exosomes released from malignant hepatocellular carcinoma (HCC) cells can increase the tumorigenic and migratory functions of low-metastatic HCC cells by inducing EMT (epithelial- mesenchymal transition), via the MAPK/ERK pathway [5], or by transferring miR-92a-3p to target PTEN and activating downstream Akt/Snail pathway [6]. Primary HCC-derived exosomes support metastases by enhancing SMAD3 signaling in circulating tumor cells to promote their adhesion [7]. Circular RNAs transferred through exosomes have also been shown to influence HCC metastasis by downregulating the miR-449a–MET pathway [8]. Similar exosome-mediated transfers of invasive and metastatic properties between cancer cells have been documented in breast cancer and ovarian cancer [9,10]. Additionally, exosomes can serve as a safe delivery system for siRNA-/shRNA-related interventions [11]. The intravenous administration of targeted exosomes can successfully deliver siRNA to the mouse brain [12]. Using orthotopic pancreatic cancer mouse models, it was demonstrated that exosomes carrying *KRAS* specific siRNA can suppress tumor growth, inhibit metastasis, and improve overall survival [13]. It remains to be determined what exosomal factors induce tumor growth and metastasis in HCC and other cancers, and whether exosomes can be exploited for targeted cancer therapy.

Recently, serum-derived and urinary exosomes have attracted much attention as an analyte in liquid biopsy for diagnosis and monitoring treatment response in cancer. Various exosomal cargoes have now been identified as candidate biomarkers for cancer diagnosis [1,14]. For example, Glypican-1 is enriched in circulating exosomes in pancreatic cancer patients and correlates with tumor burden [15]; LRG1 in urinary exosomes is a potential biomarker for detecting NSCLC [16]. Besides proteins, certain exosomal miRNAs have been correlated with poor prognosis [17]. Urinary exosomal miR-2909 was associated with prostrate cancer severity [18], while exosomal miR-141 was found to be up-regulated in patients with prostate cancer [19]. However, despite having many candidate biomarkers, few exosome-based diagnostic assays have been developed for clinical use. Ideally, finding a common exosomal biomarker for diagnosis across many cancer types would be very useful, but remains challenging. Thus far, studies have suggested that serum/plasma exosomal Glypican-1 could be a potential multi-cancer diagnostic biomarker for pancreatic, colorectal, and breast cancer [14].

Carboxypeptidase E (CPE) is an exopeptidase, initially discovered as a prohormone processing enzyme [20,21]. Subsequently, non-enzymatic functions of CPE as a sorting receptor for prohormones and a trophic factor in mediating cell survival have been reported [22,23,24]. In cancer, the aberrant upregulation of CPE is found in endocrine tumors (pituitary adenomas) [25], as well as non-endocrine tumors (cervical, colorectal, ovarian and pancreatic cancer, HCC, and glioblastoma) [26,27,28,29,30]. CPE promotes cell proliferation and migration in osteosarcoma, colorectal, and pancreatic cancer cell lines [28,31,32]. Besides the full length wild-type CPE (WT-CPE), a 40 kDa splice variant of CPE (CPE-ΔN) has been cloned and shown to promote tumor cell proliferation and invasion, by a distinct mechanism [33,34]. This 40 kDa CPE-ΔN variant is an N-terminal truncated form of the CPE protein, and is translocated into the nucleus to induce the expression of metastasis-associated genes [34]. Given the multi-faceted role of CPE in tumorigenesis, we investigated whether CPE could play a critical role in the exosomal transmission of tumorigenesis.

In this study, we investigated (1) if *CPE* mRNA and protein are present within exosomes secreted from cancer cells, and if exosomal CPE can confer the growth and metastasis of cancer cells; and (2) whether CPE-shRNA-loaded exosomes could be taken up by malignant cancer cells to inhibit tumor growth as a potential therapeutic strategy. We found that *CPE* mRNA is enriched in exosomes released from highly malignant cells of different cancer origins. Moreover, we carried out a pilot study using patient-derived sera exosomes and showed that *CPE* mRNA in circulating exosomes could be developed as a diagnostic cancer biomarker. We characterized the *CPE* mRNA and protein within exosomes from HCC cells, and showed that the down-regulation of CPE in the parental HCC97H (high-metastatic) cells prior to exosome isolation prevented the exosomal transfer of malignant properties from HCC97H to HCC97L (low-metastatic) cells. We also tested whether the exosomal route could be used to deliver CPE-shRNA to target HCC cells to inhibit proliferation, and determined the possible mechanism involved. Notably, the exosomes loaded with CPE-shRNA inhibited the growth of recipient HCC cells by suppressing Cyclin-D1 and c-MYC expression. These findings indicate that exosomal CPE and modified exosomes enclosing CPE-specific shRNA can modulate the malignant properties of cancer cells.

## 2. Results

### 2.1. Presence of CPE in Exosomes Derived from Cancer Cells

**Figure 1:**
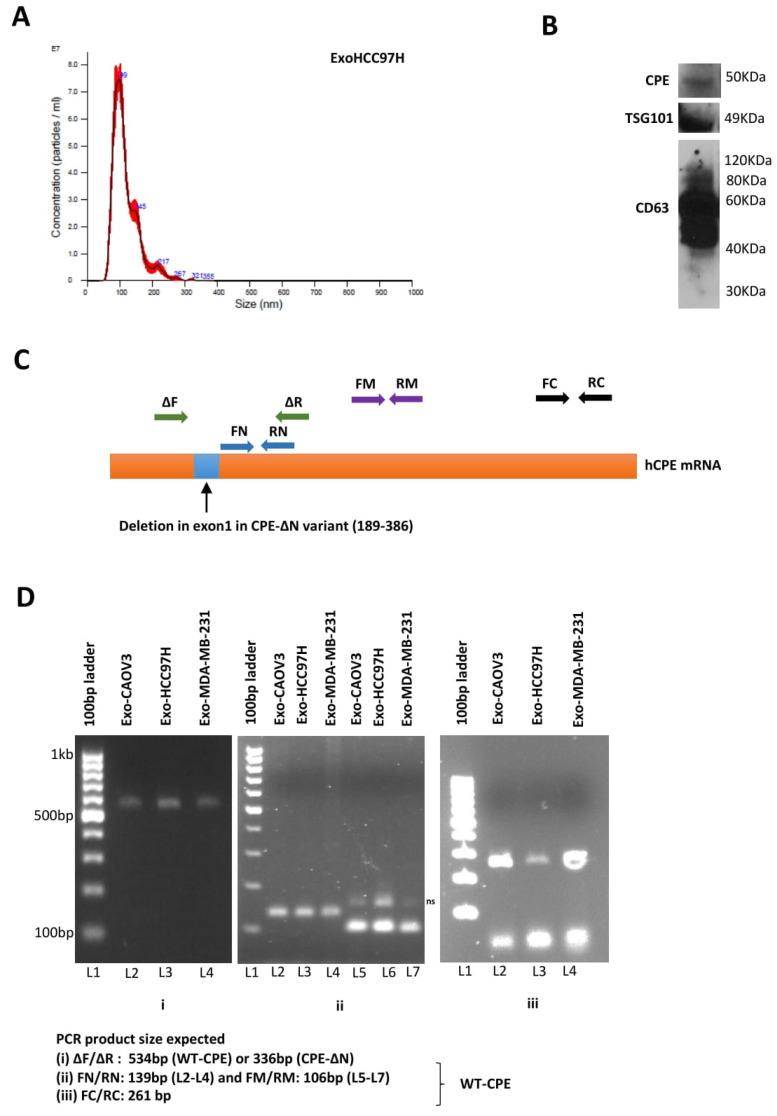
Detection of CPE in cancer cell exosomes. (**A**) Characterization of metastatic liver cancer cell derived exosomes: Representative graph (left panel) showing the concentration plotted against particle size of exosomes released from HCC97H cells, determined using NanoSight analysis. (**B**) Western blot showing WT-CPE and exosomal markers TSG101 and CD63 in exosomes released from HCC97H cells. (**C**) Schematic showing human *CPE* mRNA with the position of RT-PCR primers used to detect *CPE* gene fragments. The region of deletion seen in exon 1 of *CPE-ΔN* variant, another isoform of CPE detected in cancer cells is marked as a blue box and the ∆F/∆R primer set used to distinguish *WT-CPE* and *CPE-ΔN* sequences are denoted by green arrows. (**D**) Exosomes isolated from CAOV3, HCC97H and MDA-MB-231 cells were analyzed using RT-PCR to determine the presence of *CPE* transcripts. Images of agarose gels showing the amplicons generated using the primers specific for 5′-end (**Di**), middle (**Dii**)or 3′-end parts of *CPE* mRNA (**Diii**), besides the region flanking the exon 1 deletion in *CPE-ΔN* sequence (**Di**). The expected PCR product sizes are given below the gel images. Major band sizes represented by the 100 bp DNA ladder are shown in (**Di**). ‘ns’ refers to non-specific band in (**Dii**).

Particle analyses revealed that exosomes derived from HCC cells exhibiting high metastasis, were approximately 100 nm in diameter, as depicted in the representative graphs in Figure 1A. These vesicles were characterized by the presence of exosome-specific markers CD63 and TSG101, along with the presence of CPE (Figure 1B). The Western blot band of ~50 kDa corresponded to the size of WT-CPE (~50–53 kDa). To determine if *CPE* mRNA and its splice variant, CPE-∆N (which encodes a 40 kDa protein), are present within exosomes derived from three different cancer cell lines, we used a specific primer set ∆F/∆R which flanks the region of deletion in exon1 to differentiate *CPE-∆N* mRNA sequence, in addition to primers flanking the rest of the CPE mRNA. The primer sequences used are given in Supplementary Table S1. The position of the deletion in CPE-∆N and the primer sets used for PCR are shown in Figure 1C. As shown in Figure 1D, the amplified PCR region in exosomes derived from CAOV3 (ovarian cancer), HCC97H (liver cancer), and MDA-MB-231 (breast cancer) cell lines corresponds to WT-CPE gene segments, and not CPE-∆N. Using overlapping primer sets, we could amplify close to 1 kb from the 5′ end to the middle portion of *CPE* mRNA, while parts of 3′ region were missing, as shown in Supplementary Figure S1. Although we were unable to amplify the full-length mRNA of *CPE*, the contiguous portion of the mRNA that we amplified, in fact, encodes the entire coding sequence of *CPE* mRNA. Our results indicate that exosomes derived from HCC, breast, and ovarian cancer cell lines contain *CPE* mRNA. An analysis of HCC exosomes showed the presence of WT-CPE protein.

### 2.2. Exosomes Isolated from Highly Malignant Cancer Cells Show Elevated CPE mRNA Levels

**Figure 2:**
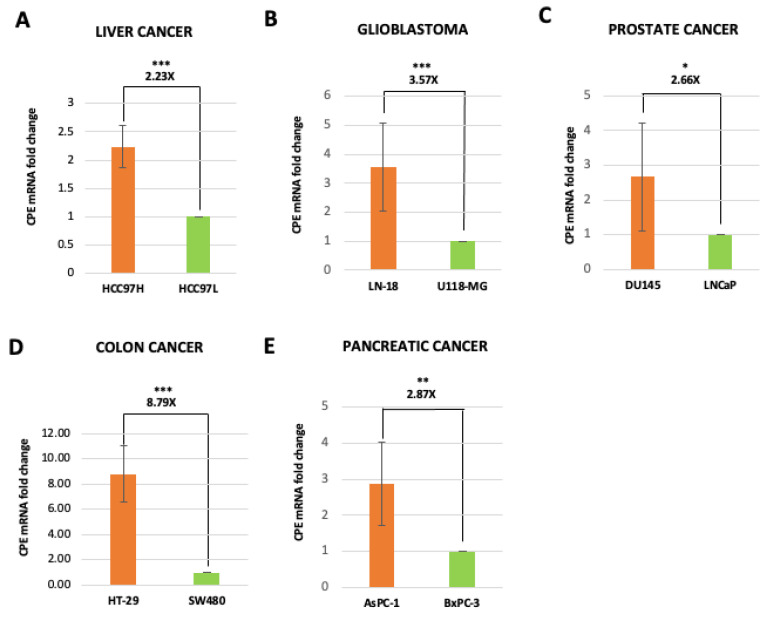
Malignant cancer cells release exosomes with elevated *CPE* copy numbers. (**A**–**E**) Bar graph showing the fold change of *CPE* mRNA copy numbers measured in exosomes derived from malignant/aggressive cells (orange bars) versus low-malignant cells (green bars) from different types of cancer as denoted in the figure (N = 3 for (**B**,**C**,**E**) and N = 2 for (**A**,**D**)). Standard curve method using CPE 5′-DNA fragment of known concentration was used to perform quantitation of *CPE* mRNA copy numbers. Data represents mean ± SD of 2 or 3 independent experiments. Error bars denote SD. Statistical analysis for all panels was performed by Student’s *t*-test: *, *p* < 0.05; **, *p* < 0.01; ***, *p* < 0.001.

Elevated expression levels of CPE have been associated with malignancy in various types of cancer cell lines in in vitro and patient tumors [26,27,28,29,31,32]. We have previously shown, using Northern blot and RT-PCR, that high-metastatic HCC97H cells have more abundant *CPE* mRNA levels compared to low-metastatic HCC97L cells [34]. Similarly, we also found that aggressive glioblastoma cells LN-18 express higher CPE mRNA levels than less aggressive U-118 cells. Previous reports further provide evidence that high-metastatic colon, prostate and pancreatic cells are associated with increased levels of CPE mRNA compared to the corresponding low-metastatic cell [32,33]. Based on these observations and our finding that cancer cell exosomes contain CPE, we then determined if the levels of *CPE* mRNA within exosomes released from these parental cancer cells (Supplementary Table S2) correlate with their malignancy. *CPE* mRNA copy numbers in the exosomes were measured using the standard curve method. Figure 2A–E shows that significantly higher *CPE* mRNA copy numbers are present in exosomes released from malignant cancer cells compared to those released from cancer cell lines with low malignancy, across various types of cancer, such as HCC, glioblastoma, prostate cancer, colon cancer, and pancreatic cancer. These data indicate that exosomes secreted by malignant cancer cells have elevated levels of *CPE* mRNA copy numbers compared to their low-malignant counterpart.

### 2.3. Serum Exosomes from Cancer Patients Have Higher CPE Transcript Copy Numbers than Healthy Controls

**Figure 3:**
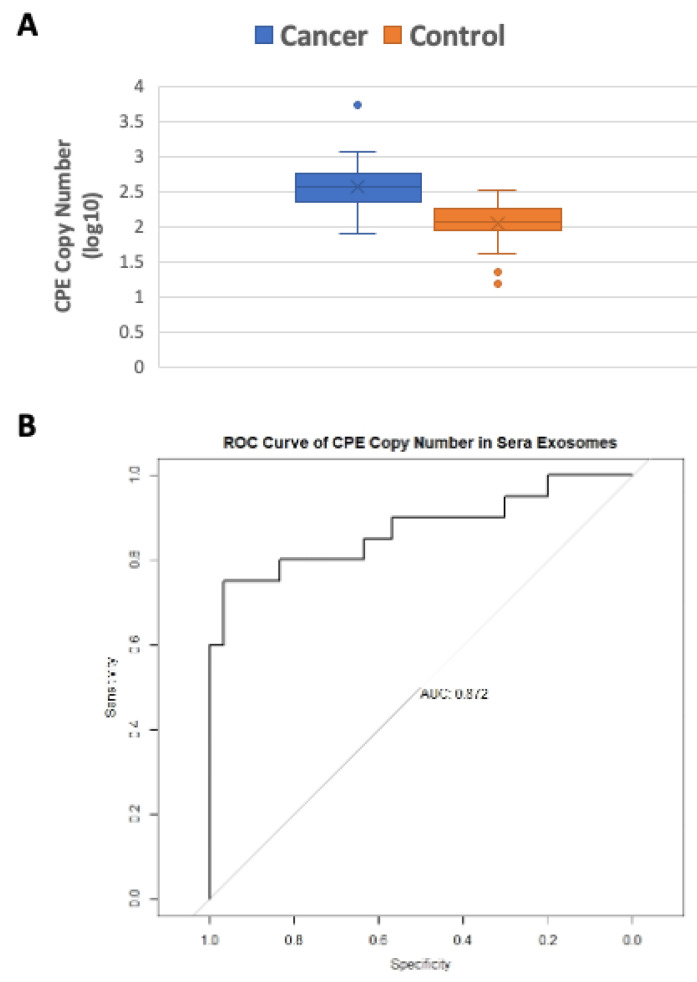
Serum exosomes from cancer patients are enriched in *CPE* mRNA. (**A**) Box plot showing the log-transformed data of CPE copy numbers in sera exosomes derived from 20 cancer patients versus 30 healthy subjects (*p* = 0.0007). (**B**) ROC curve of *CPE* copy numbers in exosomes from cancer patients’ sera compared to control sera, showing the AUC. Types of cancer included (in cases): Breast cancer (n = 5), Ovarian cancer (n = 5), Glioblastoma (n = 5), Colon cancer (n = 1), Cervical cancer (n = 1), Kidney cancer (n = 1), Pancreatic cancer (n = 1) and Prostate cancer (n = 1). Quantitation of *CPE* mRNA copy numbers was perfomed by standard curve method using CPE 5′-DNA fragment of known concentration. Logistic regression and receiver operating characteristics (ROC) curve analysis was used to determine the association of cancers with *CPE* copy number in sera exosomes.

Given that elevated *CPE* mRNA level is correlated with malignancy in cancer cell lines, we then examined the *CPE* mRNA copy number in human sera exosomes derived from patients with different types of cancer and healthy controls (see Supplementary Table S3 for subject details) in a pilot study. *CPE* mRNA copy numbers in the sera exosomes were determined using the standard curve method. The *CPE* copy numbers in serum-derived exosomes are summarized using mean (standard deviation, SD) and median (interquartile range, IQR). For the cancer cases, the mean is 670.08 (SD = 1176.98) and the median is 365.30 (IQR = 490.97−241.02 = 249.95); for the normal cases, the mean is 132.91 (SD = 72.75) and the median is 115.20 (IQR = 178.06−88.76 = 89.30). The Shapiro–Wilk normality test on the *CPE* copy number data in cancer cases showed a significant departure from normality (*p* < 0.001). Therefore, the log10 transformed data, presented in Figure 3A using box plots, are used for analysis. Logistic regression performed on the log10-transformed data showed that *CPE* copy number in sera exosomes is significantly associated with cancer (beta = 5.924, *p* = 0.0007). The empirical receiver operating characteristics (ROC) curve (Figure 3B) and its relatively large area under the curve (AUC = 0.872) corroborates the logistic regression analysis.

Box plots showing the log-transformed data of CPE copy numbers in sera exosomes from 3 major cancer types (breast cancer, ovarian cancer and glioblastoma) with n ≥ 5, compared to controls, are shown in the Supplementary Figure S2. The results from this pilot study indicate that higher *CPE* copy numbers are found in sera exosomes from cancer patients versus healthy subjects. Due to limited availability of samples, a more detailed analysis of correlation with stage/disease type has not been performed. Our current data suggest that high CPE mRNA levels in serum exosomes is indicative of cancer. This will be the basis of future research, where one can measure and compare exosomal CPE mRNA in stage-stratified patients to further explore the clinical value of its application as a biomarker.

### 2.4. HCC97H Exosomes Enhance Proliferation and Invasion of HCC97L Cells in a CPE-Dependent Manner

**Figure 4:**
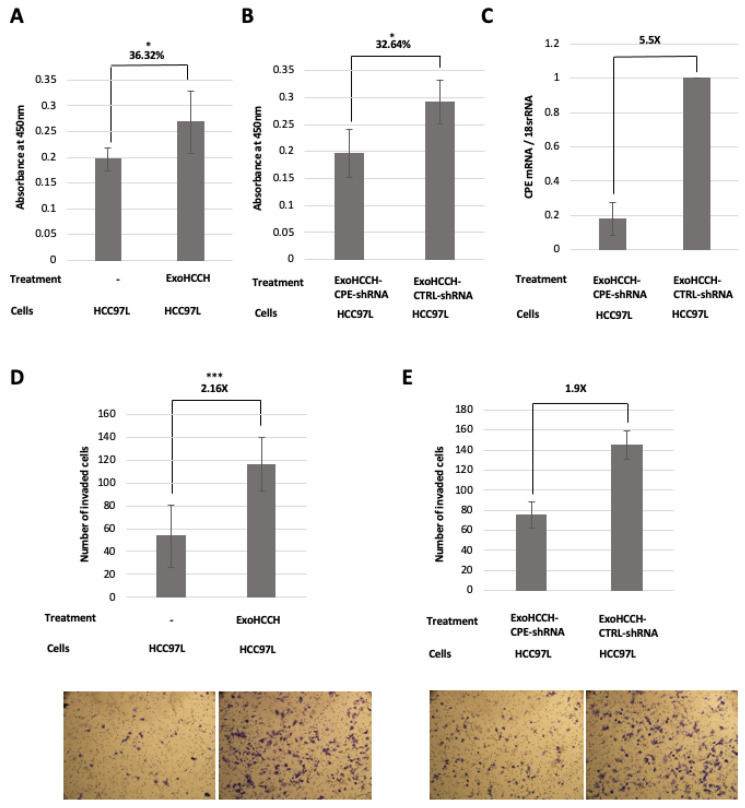
Exosomes from HCC97H cells enhance proliferation and invasion of recipient HCC97L cells in a CPE-dependent manner. (**A**,**B**) Bar graph showing the absorbance values obtained in the MTT cell proliferation assay on day 5 of HCC97L cells treated with exosomes (corresponding to 75 µg of exosomal protein) from HCC97H cells (ExoHCCH, **A**) or with exosomes isolated 48 h after lipofectamine- mediated transfection of HCC97H cells with either 25 nM of CPE targeting shRNA or control shRNA, (ExoHCCH-CPE-shRNA/ExoHCCH-CTRL-shRNA, **B**) (N = 2, n = 3). ExoHCCH increase the proliferation of HCC97L cells, however downregulation of CPE expression in HCC97H cells before exosome isolation abolishes this effect. Data represents mean ± SD of 2 independent experiments. (**C**) Bar graph showing the fold change in knockdown of *CPE* mRNA levels in HCC97L cells treated with ExoHCCH-CPE-shRNA relative to cells treated with ExoHCCH-CTRL-shRNA (N = 2). Data represents mean ± SD of 2 independent experiments. The 2−ΔΔCt method was used for gene expression analysis and 18s rRNA was the internal control. (**D**,**E**) Bar graph and representative images of wells showing the number of HCC97L cells that invaded through matrigel after treatment with ExoHCCH (**D**) (N = 2, n = 2), or with either ExoHCCH-CPE-shRNA or ExoHCCH-CTRL-shRNA (**E**) (N = 1, n = 2). Data represents mean ± SD of 2 independent experiments (**D**) and mean ± SD of technical replicates (**E**). HCCH97L cells treated with ExoHCCH exhibit enhanced invasion through matrigel, and this effect is abolished if HCC97H cells are transfected with CPE-shRNA before exosome isolation. Scale bar = 100µm. Statistical analysis for all panels was performed by Student’s *t*-test: *, *p* < 0.05; ***, *p* < 0.001.

As exosomes mediate cell–cell communication by the transfer of cargo, we investigated whether exosomal CPE taken up by recipient cells can modulate their proliferation and invasion. HCC97H and HCC97L cell lines were used as a model system to test exosomal CPE function because they exhibit high- versus low-metastatic potential respectively, and are derived from the same parental cell line [35]. We found that the incubation of HCC97L cells with HCC97H-derived exosomes increased their proliferation by ~36% (*p* = 0.03) in the MTT assay (Figure 4A) and invasion through matrigel ~2-fold (*p* < 0.0001) (Figure 4D). However, the downregulation of CPE by specific shRNA in HCC97H cells prior to exosome isolation abolished the effect of these exosomes on growth (Figure 4B, decreased by 32.64%; *p* = 0.015) and the invasion of HCC97L cells by 1.9-fold (Figure 4E). Moreover, treatment with exosomes isolated from HCC97H after the silencing of CPE expression resulted in downregulation of *CPE* mRNA levels in the recipient HCC97L cells. The gene expression was quantified using the 2−ΔΔCt method (Figure 4C). Although we used the MTT assay as a measure of cell viability, it basically indicates the metabolic activity of the cells, which could be affected by the culture conditions (e.g., media pH) and the physiological state of the cells. Nevertheless, these results indicate that exosomes isolated from HCC cells with high metastasis, when incubated with low-metastatic HCC cells, can enhance their growth and metastatic properties, and that CPE plays an important role in this process.

### 2.5. Exosomes Loaded with CPE-shRNA Inhibit Proliferation of Malignant HCC Cells

**Figure 5:**
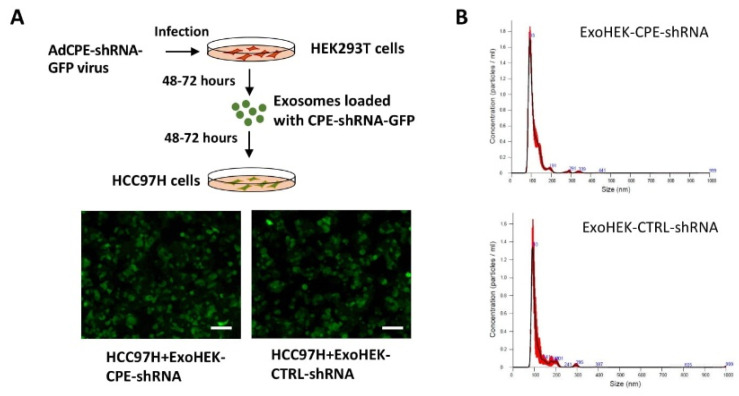
Characterization of exosomes loaded with CPE-shRNA. (**A**) Schematic showing the strategy of loading and transfer of CPE-shRNA via exosomes. Exosomes were isolated from supernatant media of HEK293T cells (ExoHEK) infected with adenovirus encoding either CPE-shRNA or CTRL-shRNA, fused to GFP. HCC97H cells treated with these modified exosomes exhibited green fluorescence, validating the transfer of CPE-shRNA through the exosomes. Representative images showing GFP fluorescence in target HCC97H cells, treated with either ExoHEK-CPE-shRNA or ExoHEK-CTRL-shRNA are included. Scale bar = 100 µm. (**B**) Graph showing the concentration and size distribution of ExoHEK-CPE-shRNA and ExoHEK-CTRL-shRNA, as determined by NanoSight analysis.

**Figure 6:**
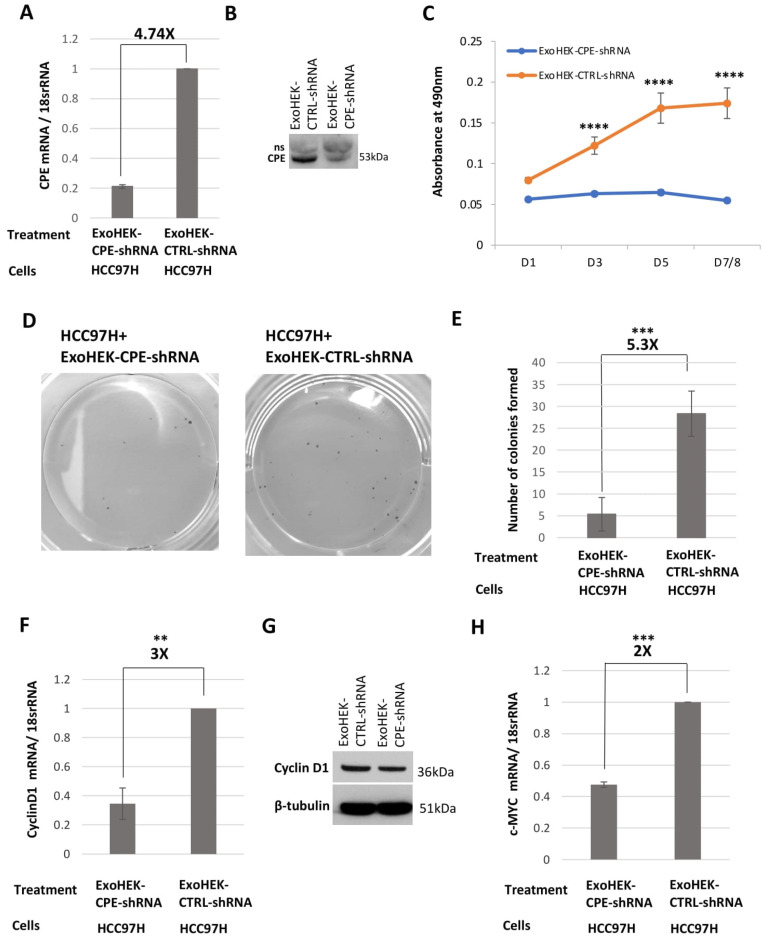
CPE-shRNA-loaded exosomes inhibit proliferation of malignant HCC cells. (**A**) Bar graph showing the fold change in downregulation of *CPE* mRNA levels in HCC97H cells treated for 48h with ExoHEK-CPE-shRNA in comparison to cells treated with ExoHEK-CTRL-shRNA (N = 2). The 2−ΔΔCt method was used for *CPE* mRNA expression analysis and 18s rRNA was used as the reference. Data represents mean ± SD of 2 independent experiments. (**B**) Western blot showing suppressed secreted CPE levels (70.91% ± 0.003 [SD] decrease) in the media of HCC97H cells treated with ExoHEK-CPE-shRNA relative to the media of cells treated with ExoHEK-CTRL-shRNA (N = 2). ns: non-specific. (**C**) Representative line graph showing the absorbance values obtained in the MTT cell proliferation assay from D1- D7/8 of HCC97H cells treated with HEK293T exosomes loaded with either CPE-shRNA or Control shRNA. CPE-shRNA loaded exosomes inhibit the proliferation of HCC97H cells (N = 3, n = 3). Data represents mean ± SD of the triplicate wells of the representative experiment. Statistical analysis was performed by Two-way ANOVA with Sidak’s multiple comparisons test. ****, *p* < 0.0001. (**D**,**E**) Representative images and bar graph showing the number of colonies formed by HCC97H cells treated with ExoHEK-CPE-shRNA or ExoHEK-CTRL shRNA. Exosomes loaded with CPE-shRNA significantly decreased the colony formation ability of HCC97H cells (N = 2, n = 3). Data represents mean ± SD of 2 independent experiments. Error bars denote SD (**F**) Bar graph showing the downregulation of *Cyclin D1* mRNA expression in HCC97H cells incubated with ExoHEK-CPE-shRNA compared to the control (N = 3). Relative qRT-PCR was performed for *Cyclin D1* mRNA quantification by 2−ΔΔCt method, and 18s rRNA was used as the reference. Data represents mean ± SD of 3 independent experiments. (**G**) Representative western blot showing reduced levels of Cyclin D1 (23.17% ± 0.022 [SD] decrease) in HCC97H cells treated with ExoHEK-CPE-shRNA compared to cells treated with ExoHEK-CTRL-shRNA (N = 2). (**H**) Bar graph showing the suppression of *c-MYC* mRNA levels in HCC97H cells after treating with ExoHEK-CPE-shRNA relative to cells treated with ExoHEK-CTRL-shRNA (N = 3). Relative qRT-PCR was performed for *c-MYC* mRNA quantification by 2−ΔΔCt method, and 18s rRNA was used as the reference. Data represents mean ± SD of 3 independent experiments. Statistical analysis for E, F and G panels was performed by Student’s *t*-test: **, *p* < 0.01, ***, *p* < 0.001.

Previous reports have shown that the injection of exosomes carrying *KRAS* siRNA could impede tumor growth and metastasis in pancreatic cancer mouse models [13]. Here, we tested if we could load HEK293T cell- derived exosomes with CPE-shRNA using adenovirus infection and then transfer the shRNA via the exosomes to target the proliferation of recipient HCC97H cells. Indeed, we detected a fluorescence signal of the GFP protein fused to the CPE-shRNA in the recipient HCC97H cells, after incubation with the exosomes isolated from HEK293T cells (ExoHEK) infected with adenovirus encoding CPE-shRNA-GFP (schematic of exosome loading and transfer is shown in Figure 5A, and the adenovirus vector map and CPE-shRNA sequence are depicted in Figure S3). These shRNA-loaded ExoHEK were characterized by NanoSight analysis and visualized using TEM, as shown in Figure 5B, Figure S4, and Table S4. No viral particles were observed in the exosome preparation, when visualized using TEM. Following treatment with ExoHEK-CPE-shRNA, a 4.74-fold reduction in *CPE* mRNA levels (Figure 6A) and a 70% reduction of secreted CPE protein (Figure 6B) were observed in the HCC97H cells, concomitant with a 3-fold decrease in cell proliferation at D7/8 (*p* < 0.0001) (Figure 6C, MTT assay) and a 5.3-fold reduction in the number of colonies formed (*p* = 0.0001) (Figure 6D,E). By co-treating HCC97H cells with AdCPE-shRNA and unloaded ExoHEK, we were able to ascertain that the growth inhibition effect seen on HCC97H cells was mediated by the transfer of CPE-shRNA by the HEK293 exosomes, and not due to any modification of exosomal content of CPE-suppressed HEK293 cells (data not shown). Furthermore, there was a 3-fold downregulation of expression of the cell cycle regulator, Cyclin D1, at the mRNA level (*p* = 0.0089) (Figure 6F), and a 23% reduction in Cyclin D1 protein (Figure 6G) in HCC97H cells treated with CPE-shRNA-loaded exosomes, consistent with the decrease in proliferation. Importantly, the expression of c-MYC, a transcription factor and proto-oncogene, was found to be significantly reduced by 2-fold (*p* = 0.0003) in the ExoHEK-CPE-shRNA-treated HCC97H cells (Figure 6H). The 2−ΔΔCt method was used for qRT-PCR-based expression analyses of CPE, Cyclin D1 and c-MYC transcripts in the HCC97H cells, with 18s rRNA as the internal control. These results show that the downregulation of CPE through exosome-mediated shRNA delivery can inhibit the proliferation of malignant liver cancer cells.

## 3. Discussion

Exosomes or extracellular vesicles are known to promote the growth and metastasis of liver and other cancers, through intercellular communication, but their internal cargo driving these effects remain unclear. Liquid biopsy assays utilizing tumor exosomes, present in many biological fluids, are being developed to diagnose and predict the prognosis of cancers such as melanoma, prostate cancer, glioblastoma and pancreatic cancer [19,36,37,38]. Serum levels of exosomal miRNAs such as miR-21, miR-141, and miR-718 have been correlated with advanced stages of squamous cell carcinoma, prostate cancer and HCC recurrence after liver transplant, respectively [19,39]. The elevated expression of CPE in tumors has been correlated with poor outcomes in patients with lung, cervical, and pancreatic cancer, and hepatocellular carcinoma [27,28,29,40]. Furthermore, CPE has been shown to promote the survival, growth, and invasion of tumor cells [26,28,32,41,42]. We therefore investigated whether CPE is present within cancer cell exosomes, and if so, if it plays a pivotal role in promoting tumor cell proliferation and invasion in recipient cells. Indeed, we found *CPE-WT* mRNA, but not the *CPE-ΔN* variant within the exosomes derived from liver, breast and ovarian cancer cells. Interestingly, the contiguous portion of the mRNA (~1.2 kb) that we detected encodes the entire coding region of *CPE*, with some of the noncoding 3′ end missing. Full-length *CPE* in HCC and other cancer cells is ~2.4 kb [34], but whether this 1.2 kb transcript of *CPE* mRNA could be successfully translated to yield a functional protein awaits future studies. Within the exosomes derived from HCC97H cells, we found a ~50 kDa CPE protein approximating the size reported for WT-CPE. These data reveal that both *CPE* mRNA and protein are packaged inside cancer cell exosomes.

Consistent with reports that elevated CPE expression levels in tumors correlate with the progression of the disease [26,27,28,29,31,32], we demonstrated that *CPE* mRNA copy numbers are significantly higher in exosomes isolated from malignant cancer cells compared to low-malignant cancer cell exosomes, across different cancer types. The finding of a positive correlation of *CPE* mRNA copy numbers with malignancy suggests that circulating exosomal CPE could potentially serve as a useful biomarker to detect cancer in patients. To this end, as a proof of concept, we showed that significantly high *CPE* mRNA copy numbers are present in serum-derived exosomes from patients with various types of cancer versus normal healthy controls. However, while the results are promising, this remains a pilot clinical study, as the sample size is small, and extensive studies with more patients with different cancer types are necessary to develop the use of exosomal CPE as a cancer biomarker.

Accumulating evidence suggest that transfer of exosomal cargo is linked to cellular communication within the tumor microenvironment and metastatic disease development. Exosomes from highly metastatic melanoma ‘educate’ bone marrow progenitors by elevating their MET receptor (hepatocyte growth factor receptor) expression, thereby facilitating primary tumor growth and metastasis [4]. Previous studies have shown that it is possible to transfer the metastatic behavior of highly malignant cancer cells to those with low malignancy through exosomes [43]. We have previously shown, by Northern blot analysis, that CPE mRNA levels in HCC97L cells are extremely low, when compared to HCC97H cells [34], and hence, we used HCC97L cells to examine if CPE could be potentially involved in the phenotypic transformation of these cells on treatment with exosomes secreted by HCC97H cells. Our study demonstrated that both the proliferation and invasion of HCC97L cells were significantly increased by incubation with HCC97H exosomes. Most importantly, we showed that this phenocopying of malignant behavior in HCC cells via exosomes was dependent on CPE. Thus, our data indicate that the exosomal cargo, CPE, plays a key role in exosome-mediated cell–cell communication to promote liver cancer proliferation and invasion. Future research will determine the mechanism of how exosomal CPE mRNA/protein derived from HCC97H cells mediates the tumor enhancing effect in HCC97L cells. As we stated in our previous publication [34], CPE is primarily secreted in HCC and other cancer cell lines. It is therefore difficult to detect and quantify CPE protein in cancer cell extract, as it is rapidly secreted after biosynthesis. This poses a challenge for quantifying any increase in CPE protein levels, in the ExoHCCH-treated HCC97L cells. In addition, we do not yet understand how the CPE mRNA/protein in exosomes is taken up by the recipient cells or the CPE protein’s intracellular route and fate after uptake. Similarly, the exact mechanism of how the silencing of CPE expression in the HCC97H cells, prior to exosome isolation, blocks the pro-tumorigenic effect on the HCC97L cells is also not clear. It could be through the modulation of intrinsic cell properties of HCC97H cells, which later impact the exosomal content, and not necessarily a direct effect of CPE content in the exosomes. This speculation can also be extended to the observation that the treatment of HCC97L cells with ExoHCCH induces tumor enhancing effects by way of either the transfer of CPE mRNA/protein to the recipient HCC97L cells or by other CPE-regulated target genes/proteins present within the milieu of the HCC97H derived exosomes. The suppression of CPE in the exosome producer HCC97H cells clearly abolishes the tumor enhancing effects on low-metastatic HCC cells, strongly supporting that CPE is important for exosome-mediated malignant transformation. Interestingly, we observed that CPE mRNA levels are downregulated in the HCC97L cells when incubated with exosomes derived from CPE-shRNA treated HCC97H cells, but not when treated with control-shRNA. This result suggests that the suppression of CPE mRNA expression in the recipient cells after exosome treatment could have caused the repressive effects on proliferation and invasion.

As we found correlation of elevated *CPE* mRNA levels with high malignancy in many other cancer cells, including breast cancer, prostate cancer, pancreatic cancer, and glioblastoma, we speculate that exosomal CPE could also likely promote the proliferation and invasion of these cancer types. The mechanism by which exosome associated CPE transfers the malignant phenotype to recipient cells requires more investigation.

Exosomes have been shown to act as vehicles to safely deliver cargo such as siRNA to the brain and pancreas [12,13]. We showed that CPE-shRNA transferred via exosomes to HCC97H cells can downregulate their tumorigenic propensity, through the suppression of Cyclin-D1 and c-MYC levels. In general, the over-expression of Cyclin D1 is associated with tumor progression, chemotherapeutic resistance, and metastasis [44,45], while the upregulation of c-MYC, a transcription factor that regulates proliferation and cell-cycle progression, is strongly correlated with poor prognosis in liver cancer patients, including metastasis [46]. p53 mutations, when combined with the constitutive activation of c-MYC, can lead to DNA damage and induce liver tumorigenesis [47]. Indeed, earlier reports have suggested that Cyclin D1 acts downstream of CPE in colorectal cancer and osteosarcoma cells, to promote the proliferation of these cells [7,31,32]. c-MYC was identified as one of the genes that showed 2-fold downregulation in HCC97H cells treated with ExoHEK-CPE-shRNA versus ExoHEK-CTRL-shRNA, using a Human Tumor Metastasis −RT^2^ Profiler PCR Array (QIAGEN, Cat# 330231 PAHS-028ZA; data not shown), and hence, was chosen for further validation in this study. We propose that CPE controls its targets, such as c-MYC and Cyclin D1, through binding a receptor to activate downstream signaling. We have recently found that CPE activates a receptor HTR1E to activate the ERK pathway [48]. ERK/c-MYC and ERK/Cyclin D1 signaling are well known in promoting proliferation and migration in cancer cells, and HTR1E has been found in human cancer cells. This is one possible way for secreted soluble CPE to promote tumor cell growth, although CPE may activate other signaling pathways to regulate cancer growth and metastasis [49]. At the present time, we do not know how exosomes which release their cargo, including CPE into the cytoplasm of the cell, activate their downstream targets. However, extrapolating from our studies of 40kD CPE-ΔN, the splice variant lacking the N-terminus signal peptide, which does not go into the RER/Golgi secretory pathway, but is translocated from the cytoplasm into the nucleus where it acts as a transcription factor to activate many genes including β-catenin, c-MYC and Cyclin D1 [50], we speculate that exosomal WT-CPE released into the cytoplasm could up-regulate c-Myc and Cyclin D1 expression in a similar manner. Our results highlight the potential of exosomes harboring CPE-shRNA to be developed as a therapeutic agent for treating HCC. Interestingly, treatment with exosomes carrying shRNA to target *KRAS* has suppressed tumor progression and enhanced survival in pancreatic cancer mouse models [13]. A similar strategy using CPE-shRNA loaded exosomes could also be applied to other tumors such as glioblastoma, osteosarcoma, colorectal cancer, and pancreatic cancer, where CPE plays a pro-tumorigenic role.

## 4. Materials and Methods

### 4.1. Cell Culture

Human cancer cell lines HCC97H, HCC97L (liver cancer); MDA-MD-231, MCF-7 (breast cancer); AsPC-1, BxPC-3 (pancreatic cancer), HT-29, SW480 (colorectal cancer), DU145, LNCaP (prostate cancer) and LN-18, U118-MG (glioblastoma), exhibiting either malignant or low-malignant potential, respectively, and malignant CAOV3 cells (ovarian cancer), were cultured in DMEM medium supplemented with 10% fetal bovine serum at 37 °C in a humidified 5% CO_2_ incubator. The various cancer cell lines were seeded at approximately equal numbers in the culture dish and maintained at similar conditions, such as volume of growth media and incubation time. All cell lines, except HCC cells, were obtained from ATCC (Manassas, VA, USA). Human HCC cell lines with low- and high-metastatic potential, MHCC97L and MHCC97H (referred to in this study as HCC97L or low-metastatic HCC and HCC97H or high-metastatic HCC), respectively, derived from the same parental cell line, were obtained from Liver Cancer Institute, Fudan University (Shanghai, China).

### 4.2. Patient Serum Samples

Blood samples were collected from 22 patients diagnosed with different types of cancers prior to surgery, and the serum was prepared and stored at −80 °C till exosome isolation. Sera were obtained from glioblastoma patients diagnosed with WHO Grade IV Glioblastomas (IDH wild type) prior to surgery, from UCSD Medical Center, San Diego, CA (IRB 120345). All other cancer serum samples were from patients with Stage I and II tumors, except 2 stage III (colon and ovarian), 2 benign (breast and colon), 1 unknown (ovarian) and 2 invasive but stage not known (breast), and were obtained from Maine Medical Center BioBank (Portland, ME, USA), which operates under an Institutional Review Board (IRB) approved protocol, and is overseen by the MMCRI Office of Research Compliance (FWA00003993). Sera from 30 healthy donors were obtained at the National Institutes of Health from The Blood Bank and under protocol 00-CH-0093, approved by IRB of the Eunice Kennedy Shriver National Institute of Child Health and Human Development, Bethesda, MD, USA. All serum samples were coded and unidentified.

### 4.3. Isolation of Exosomes

When cells seeded in a 60 mm dish reached 75% confluency (~2.5 × 10^6^ cells), the supernatant media were collected and pre-cleared of cell debris by centrifugation at 2500 rpm for 10 min at 4 °C. Exosomes were isolated from the pre-cleared supernatant culture media of cells using ExoQuick TC reagent (System Biosciences, EXOTC50A-1, Palo Alto, CA, USA), according to manufacturer’s instructions. Briefly, 1 mL of reagent was added per 5 mL of culture media, and incubated at 4 °C for at least 12 h. Exosomes present in the incubated media were then pelleted down by centrifugation at 1500× *g* for 30 min and resuspended in either 50 µL of PBS or TRIzol reagent (Sigma, St. Louis, MO, USA) for RNA isolation or in RIPA protein lysis buffer for Western blot, and stored in −80 °C until further use. Serum exosomes were isolated from 250 µL serum using ExoCap composite kit (MBL International, Woburn, MA, USA) per instruction manual, which is based on an antibody coupled magnetic capture bead-based procedure. The kit contains a mixture of CD9, CD63, CD81 and EpCAM capture beads. This step was followed by the purification of exosomal RNA using ExoCap Nucleic acid elution buffer (MBL International, MEX-E kit, Woburn, MA, USA), according to the kit protocol.

### 4.4. NanoSight Analysis

A nanoparticle tracking analysis (NTA) was performed to determine size distribution and concentration of exosomes using NanoSight LM10 instrument (Malvern Panalytical, Malvern, UK), equipped with a 405 nm LM12 module and EM-CCD camera (DL-658-OEM-630, Andor Technology, Belfast, UK) and NTAv3.1 software (Malvern Panalytical, Malvern, UK). Two microlitres of exosomes were diluted with 500 µL of PBS before analysis. The dilution factor was accounted to obtain the final exosome concentrations. Results are displayed as a graph with size (nm) vs. concentration (particles/mL) measurements, and a scatter plot with size (nm) vs. intensity (a.u).

### 4.5. RT-PCR

cDNA was synthesized from 3–6 µg of RNA from exosomes using sensiFAST cDNA synthesis kit (BIOLINE Meridian Bioscience, BIO-65053, Memphis, TN, USA) based on manufacturer’s instructions. CPE transcript was amplified using SeqAMP DNA polymerase (Clonetech, catalog no: 638509, Mountain View, CA, USA) and different primer sets, as indicated in the corresponding figure. Primer sequences are given in Supplementary Table S1. The PCR cycle consisted of an initial ‘hot start’ at 94 °C for 3 min, followed by 35 cycles of amplification (94 °C 30 s, 60 °C 30 s, 72 °C 30 s), with a final extension step of 72 °C for 5 min. PCR products were analyzed on 1.8% agarose gels.

### 4.6. Quantitative Real-Time PCR

Exosomal RNA was purified from supernatant media of cells using SeraMir kits (System Biosciences, RA800A, Palo Alto, CA, USA) or TRIzol reagent (Sigma-Aldrich, St. Louis, MO, USA), and from serum using ExoCap composite kits. TRIzol isolated RNA from exosomes was used only for RT-PCR experiments shown in Figure 1C,D and Supplementary Figure S1. The first-strand cDNA was synthesized with 0.1 µg of total RNA using SensiFast cDNA Synthesis kit (BIOLINE Meridian Bioscience, Memphis, TN, USA). qRT-PCR was performed using SYBR Green PCR Matrix Mix (Applied BioSystem, #4367659, Waltham, MA, USA) in an ABI PRISM 7900 Sequence Detector (Applied Biosystems, Waltham, MA, USA), with cycling conditions as: 95 °C for 5 min, followed by 40 amplification cycles of denaturation 95 °C for 15 s, annealing 60 °C for 60 s, and extension 72 °C for 30 s, and final extension at 72 °C for 10 min. In the absence of a good internal control for exosomal mRNA normalization, the standard curve method using a CPE 5′-DNA fragment of known concentration was used to perform quantitation of *CPE* mRNA copy numbers in exosomes using FN/RN primer set. All samples for sera copy number determination including the standard curve were run together in a 384-well PCR plate. For cancer cell exosomes, the fold change in exosomal CPE mRNA copy number of high-metastatic cells with respect to the exosomal CPE mRNA copy number of low-metastatic cells was determined by dividing the first number with the latter. The mean fold change ± SD of the independent experiments is shown in the bar graph. This was done across the different cancer types. TRIzol was used to isolate RNA from HCC cells. The 2−ΔΔCt method was used to calculate the relative fold difference of mRNA expression of *CPE*, *Cyclin D1*, and *c-MYC* in HCC97L and HCC97H cells. 18s rRNA was used for data normalization. Primer sequences used are listed in Supplementary Table S1. All qRT-PCR assays were run in triplicate.

### 4.7. Western Blot

Exosome/cellular protein lysates were prepared using RIPA lysis and extraction buffer (Thermo Fisher Scientific, #89901, Waltham, MA, USA) supplemented with Halt Protease inhibitor cocktail (Thermo Scientific, #87786, Waltham, MA, USA). Forty-five µg of exosomal protein or 25 µg of cellular protein was loaded per lane of the SDS-PAGE gel, and Western blot was performed, as described previously [42]. For the analysis of secreted WT-CPE, the supernatant media of cells were concentrated using Amicon Ultra 10k MWCO centrifugal filter (Millipore Sigma, St. Louis, MO, USA). Monoclonal antibody against CPE (#610758, 1:2000 dilution) was purchased from BD Biosciences (Franklin Lakes, NJ, USA), and primary antibodies to TSG101 (ab612696, 1:500 dilution) and CD63 (ab68418, 1:1000 dilution) were from Abcam (Cambridge, MA, USA). Cyclin D1 (#92G2, 1:500) antibody was from Cell Signaling Technology (Danvers, MA, USA) and β-tubulin (#T5168, 1:2000) was procured from Sigma-Aldrich (St. Louis, MO, USA).

### 4.8. In Vitro Exosome Transfer Experiments

To perform exosome transfer experiments using HCC97H-derived exosomes, HCC97H cells were seeded in a 60 mm dish and transfected with either 25 nM CPE-shRNA, which is a pool of three target-specific lentiviral vector constructs (each encoding 19–25 nt shRNAs) or control shRNA plasmids (Santa Cruz Biotechnology Inc, Cat#sc-45378-SH, sc-108060, Dallas, TX, USA) using Lipofectamine 2000 reagent (Thermo Fisher Scientific, Waltham, MA, USA). Forty-eight hours later, the supernatant media of the transfected cells were collected, and exosomes were isolated. Exosomes were also isolated from the culture media of untransfected HCC97H cells (ExoHCCH) for some experiments. After dissolving the exosome pellet in 50 µL of PBS, the exosomal protein was estimated using protein assay (Bio-Rad Laboratories, Cat#500-0006, Hercules, CA, USA). HCC97L cells seeded in a 6-well plate were treated with 75 µg of exosomal protein/well for 48 h, after which the cells were harvested, and seeded for MTT and cell invasion assays. Based on the NanoSight analyses of ExoHCCH, ExoHCCH-CPE-shRNA and ExoHCCH-CTRL-shRNA, the number of particles added was quantitated to be approximately equal to 55–70 × 10^10^ particles/well.

For experiments targeting HCC97H with CPE-shRNA-loaded exosomes, HEK293T cells were infected with adenovirus carrying either CPE-shRNA-GFP or control-shRNA-GFP (Vector Biolabs, Cat# shADV-229236, Malvern, PA, USA) at MOI 25 for 48–72 h. After 5–6 h of infection, the culture media were replaced to remove viral particles present in the infection media. Exosomes were isolated from the supernatant media of the infected cells, and the exosomal protein was estimated. To compare and standardize exosome loading, 25 µg of the exosomal protein (exoHEK), either exoHEK-CPE-shRNA or exoHEK-CTRL-shRNA, were used to treat HCC97H cells, seeded in 4-well chamber slides. Moreover, 48 h later, the GFP (green fluorescent protein) fluorescence of the cells, which is an indirect measurement of shRNA loading and transfer via exosomes, was documented using a fluorescent microscope (Eclipse 80i, Nikon or Zeiss Wide-Field), and the GFP levels were quantitated using Image J software using the following formula:

CTCF (corrected total cell fluorescence) = Integrated Density − (Area of selected cell × Mean fluorescence of background readings)

Area, mean fluorescence, and integrated density values are obtained from the Image J software (http://imagej.nih.gov/ij/, accessed on 15 March 2019). The fold change difference in the GFP levels between ExoHEK-CPE-shRNA and ExoHEK-CTRL-shRNA treated HCC97H cells, if any, is determined. Subsequently, HCC97H cells seeded in a 30 mm dish were treated with 100 µg of ExoHEK-CPEshRNA. The amount of ExoHEK-CTRL-shRNA to be added was calculated based on the fold change difference in the GFP levels, determined by Image J software analysis of fluorescent images, performed in the prior standardization step, such that the GFP levels between the ExoHEK-CPE-shRNA and ExoHEK-CTRL-shRNA treatment groups are comparable. After 48 h, the cells were seeded for MTT and colony formation assays.

### 4.9. Cell Proliferation Assay

To assess the proliferation of cells, 2000 cells/well were seeded in a 96-well plate and the MTT (3-(4,5-dimethylthiazol-2-yl)-2,5-diphenyltetrazolium bromide) assay was performed on days 1, 3, 5 and 7/8, as reported previously [51]. Absorbance reading was taken at 490 nm or 450 nm in a microplate reader (BioTek, Winooski, VT, USA).

### 4.10. Matrigel Invasion Assay

Furthermore, a 24-well Corning Matrigel invasion chamber (Corning, NY, USA) with 8-μm pores was used to perform the cell invasion assay. Briefly, 500 µL of cell suspension (1 × 10^5^ cells/mL) in serum-free media was added to the top chamber, while serum supplemented media were added to the lower chamber. After 24 h, invaded cells were fixed with 100% methanol and stained with 1% crystal violet solution. Images from five different fields/well were captured, and cells were counted.

### 4.11. Colony Formation Assay

Cells were seeded in a 6-well plate at a density of 2000 cells/well and cultured for 11–15 days to allow colonies to form, following which, they were fixed using 100% methanol and stained with 1% crystal violet solution. Representative images of wells were taken, and a number of colonies containing at least 50 cells were counted using Image J software (http://imagej.nih.gov/ij/, accessed on 15 March 2019).

### 4.12. Statistical Analysis

The data represent mean ± SD (standard deviation) of independent experiments (N), performed in triplicate (n = 3), or as stated in the figure legend. Statistical significance was determined using Student’s *t*-test or two-way ANOVA, and p values are denoted as * *p* < 0.05, ** *p* < 0.01, *** *p* < 0.001 and **** *p* < 0.0001, and are specified in the figure legend. A two-way ANOVA with Sidak’s multiple comparisons test was performed using GraphPAD PRISM. Box plot and Shapiro-Wilk normality test were used to examine the distribution of *CPE* copy numbers in human sera exosomes. A logistic regression and a receiver operating characteristics (ROC) curve analysis were performed to investigate the association of cancers with *CPE* copy number in sera-derived exosomes.

## 5. Conclusions

We have identified a new bioactive molecule, CPE, in exosomes, that has the ability to transfer the malignant phenotype from low- to high-metastatic HCC cells, suggesting that circulating exosomes carrying CPE may represent a novel mechanism for promoting tumor metastasis in the body. Our data show that exosomes modified to carry CPE-shRNA could suppress tumor growth and be a potentially exciting new therapy for treating liver and other cancers, since CPE expression is upregulated in many cancer types. Our pilot clinical study suggests that *CPE* mRNA in circulating exosomes could be developed as a biomarker for diagnosing cancer. Future investigations will focus on translating our findings to pre-clinical models and advancing the potential clinical use of the exosome-based delivery of CPE-shRNA in the treatment of different types of cancer.

## Data Availability

Data supporting the findings of this study are contained within the article and the additional supplemental files. All data discussed in the paper and all materials will be made available to readers upon request from the corresponding author.

## Supplementary Materials

The following supporting information can be downloaded at: https://www.mdpi.com/article/10.3390/ijms23063113/s1.

## Abbreviations

CPE	Carboxypeptidase E
HCC	Hepatocellular carcinoma
HEK	Human embryonic kidney
TSG101	Tumor susceptibility gene 101
AUC	Area under the curve
ROC	Receiver operating characteristics
GFP	Green fluorescent protein
NTA	Nanoparticle tracking analysis
EMT	Epithelial-Mesenchymal Transition
