# Supplementary material for: Exosomal Carboxypeptidase E (CPE) and CPE-shRNA-Loaded Exosomes Regulate Metastatic Phenotype of Tumor Cells
Source: Int J Mol Sci. 2022 Mar 14;23(6):3113. doi: 10.3390/ijms23063113 (PMC8953963; doi:10.3390/ijms23063113)
Supplement: Supplementary file 1 [file ijms-23-03113-s001.zip › IJMS full gel blots- with edges, 11-23-21.pdf]

Full gel blots of Figure 1B

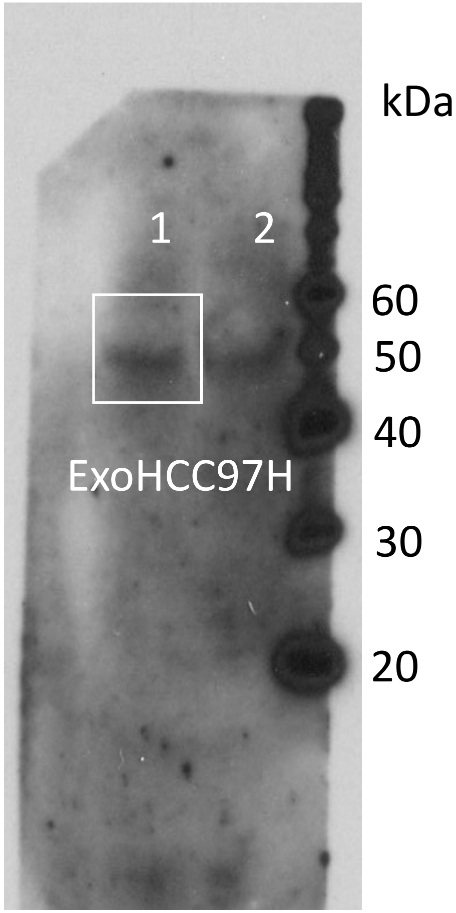

CPE Blot

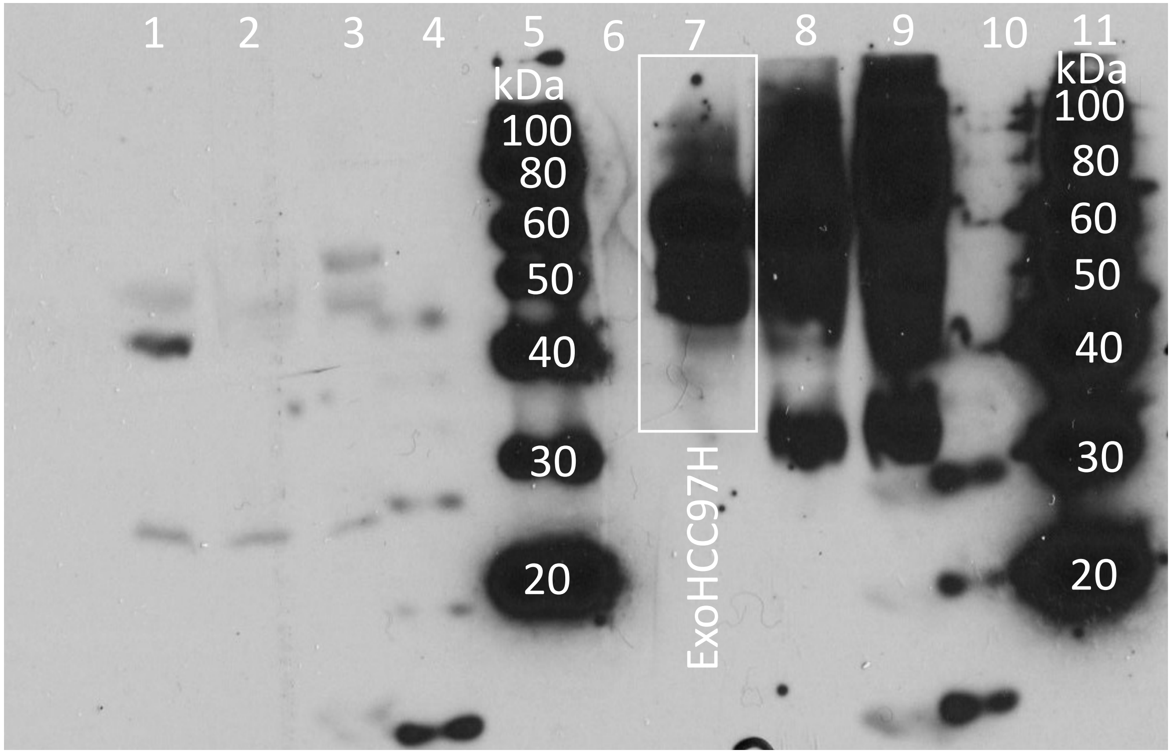

CD68 Blot

Full gel blot of Figure 1B

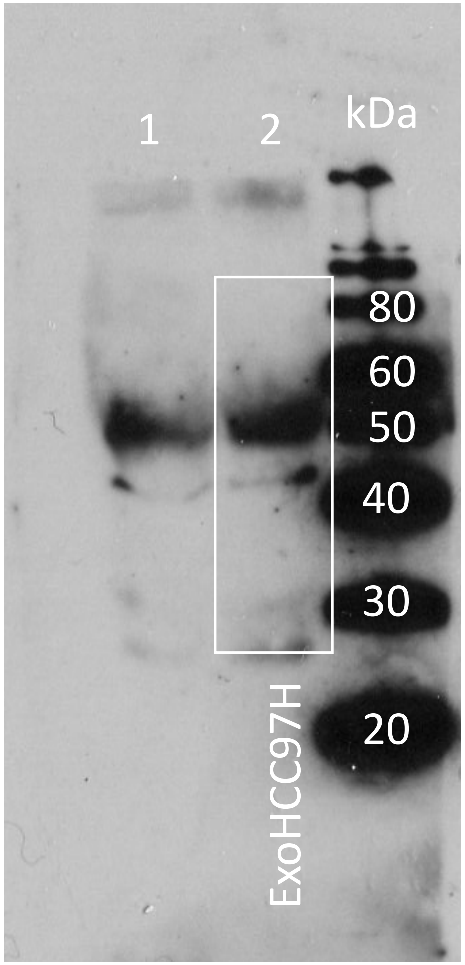

TSG101 Blot

Full gel blot of Figure 6B

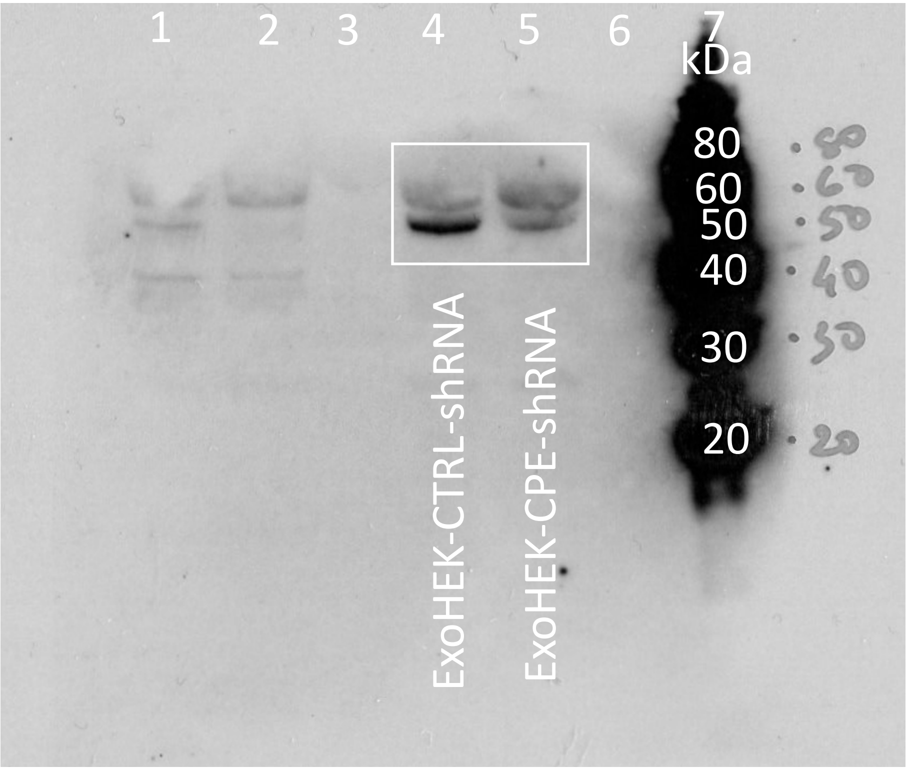

CPE Blot

Full gel blot of Figure 6G

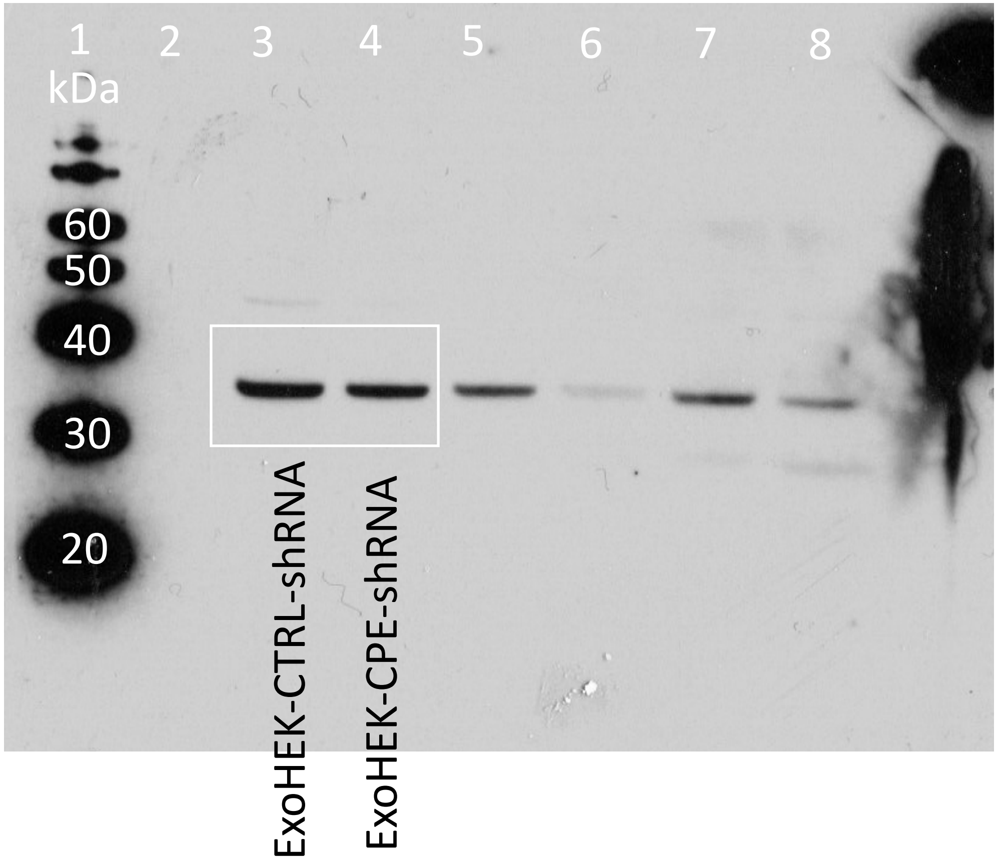

Cyclin-D1 Blot

Full gel blot of Figure 6G

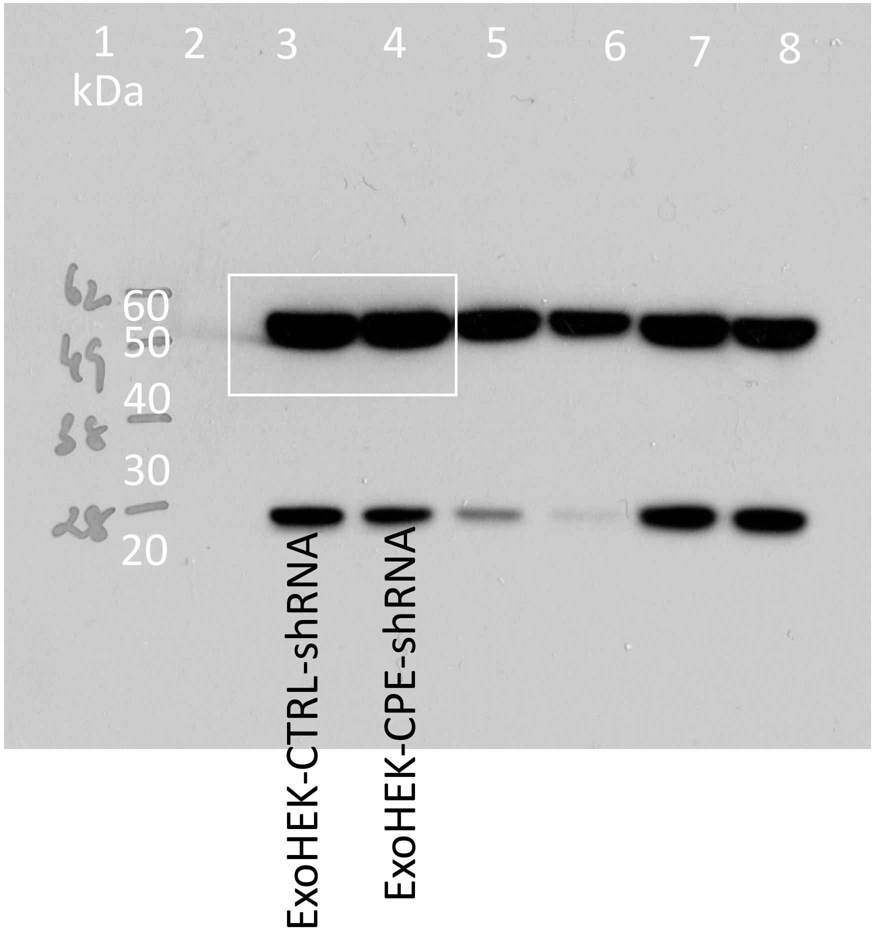

β -tubulin blot (Cyclin-D1 blot was stripped and reprobed for β -tubulin)
