# Supplementary material for: Exosomal Carboxypeptidase E (CPE) and CPE-shRNA-Loaded Exosomes Regulate Metastatic Phenotype of Tumor Cells
Source: Int J Mol Sci. 2022 Mar 14;23(6):3113. doi: 10.3390/ijms23063113 (PMC8953963; doi:10.3390/ijms23063113)
Supplement: Supplementary file 1 [file ijms-23-03113-s001.zip › IJMS Suppl File- revised round 2 final, 02-10-22.pdf]

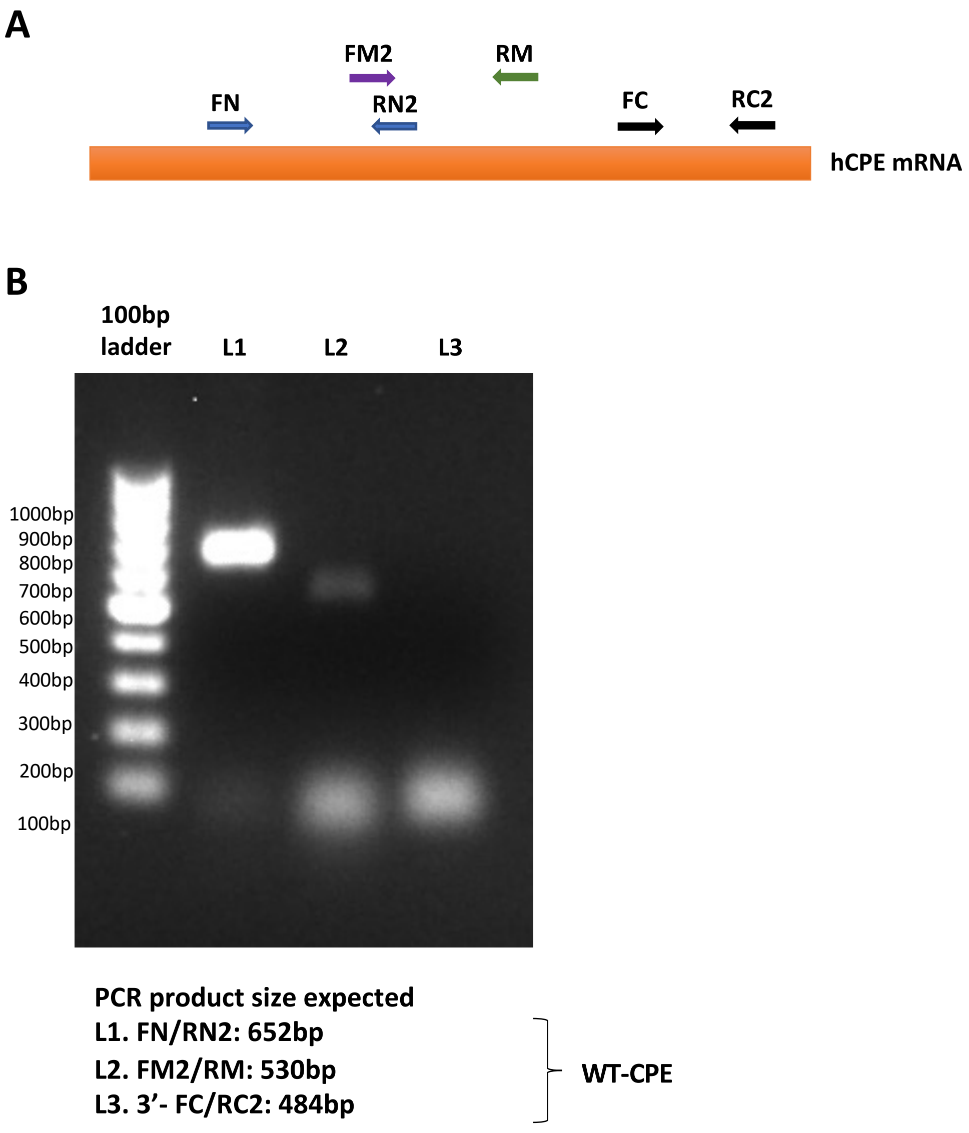

Figure S1. Detection of CPE mRNA in cancer cell exosomes. (**A**) Schematic showing human *CPE* mRNA with the position of overlapping RT-PCR primer sets FN/RN2 and FM2/RM, which cover 5' end to the middle portion of *CPE* mRNA, and a different set of 3'primers, FC/RC2. **(B)** Exosomes isolated from HCC97H cells were analyzed using RT-PCR using the primers shown in a. Image of agarose gel showing the amplicons generated and the expected PCR product sizes are shown. Using the overlapping primer sets, amplification from 5'end until the middle portion of CPE mRNA was achieved, however the 3' primers failed to generate the corresponding PCR product, indicating that the 3' end of *CPE* mRNA in HCC97H exosomes is fragmented.

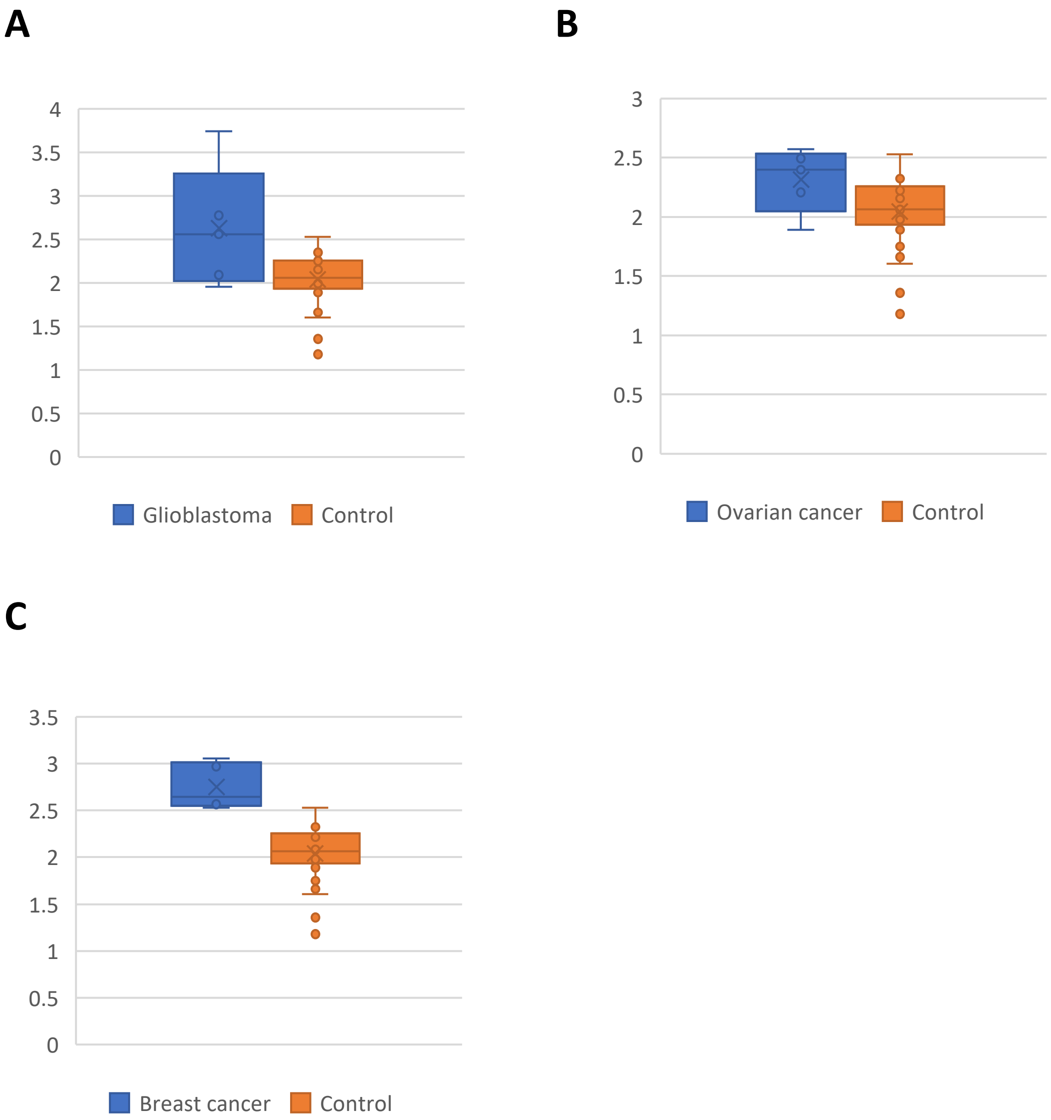

Figure S2. *CPE* mRNA is enriched in serum exosomes from cancer patients. **(A-C)** Box plot showing the log-transformed data of *CPE* copy numbers in sera exosomes from 5 glioblastoma, 5 ovarian cancer and 5 breast cancer patients versus 30 healthy subjects.

A

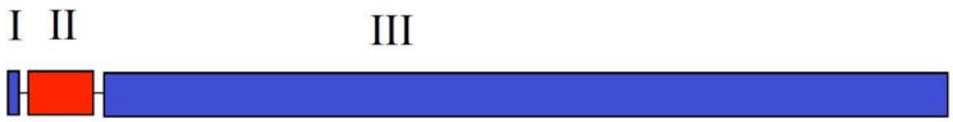

**I:**	**Human Ad5 sequences (hAd5wt 1-458; includes 5' L-ITR and packaging signal)**
**II:**	**U6-shRNA-CMV-eGFP-PolyA**
**III:**	**Human Ad5 sequences (hAd5wt 3513-35935; E3 region deleted, includes 3' R-ITR)**<br>**E3 deletion: 28587 - 30464**

B

5'- CCGG-CTCCAGGCTATCTGGCAATAACTCGAGTTATTGCCAGATAGCCTGGAG-TTTTT-3'

Figure S3. Details of the adenovirus shRNA construct **(A)** Schematic map of adenovirus construct (30, 000bp). U6 promoter drives the expression of the transgene while the eGFP reporter is under a CMV promoter. (B) The sequence of the CPE-shRNA, inserted into the vector. *Source: Vector Biolabs.*

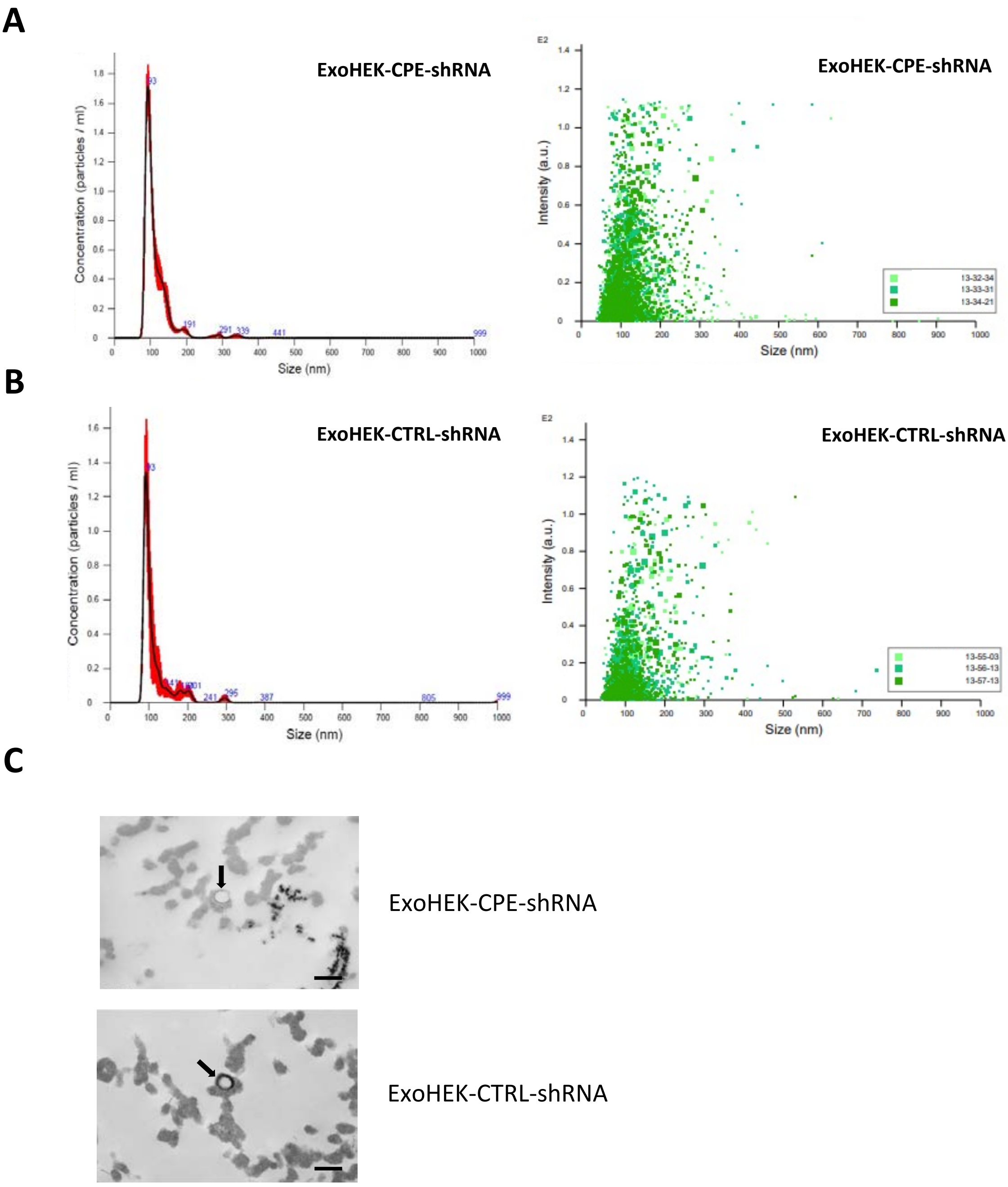

Figure S4. Characterization of CPE-shRNA or Control-shRNA loaded exosomes from HEK293T cells. **(A-B)** Representative graph (left panel) showing the concentration plotted against particle size and scatter plot (right panel) indicating size vs intensity of ExoHEK-CPE-shRNA (A) and ExoHEK-CTRL-shRNA (B), as determined by NanoSight analysis. **(C)** Representative TEM images of exosomes (see arrows) loaded with either CPE-shRNA or CTRL-shRNA. Scale bar= 400 nm.

Table S1. Sequences of the PCR primers.

Primer ID	Forward primer	Reverse primer
ΔF/ΔR	CATTCAGCCGGGGAAGGT G	GTACTGGGCCAAGAAAATGAGC
FN/RN	GCCGGCGGCTGCAGCAAG	GCTGCGCCCCACCGTGTAAATC
FM/RM	CCATCTCCGTGGAAGGAATA	CCTGGAGCTGAGGCTGTAAG
FC/RC	AGCTCGGAGTTGTGAGCACTCTA	GGCCACTCTACAACCAAGAGAAA
FCyc/RCyc	AACTACCTGGACCGCTTCCT	CCAC TTGAGCTTGTTCACCA
F18s/R18s	CTCTTAGCTGAGTGTCCCGC	CTGATCGTCTTCGAACCTCC
FMyc/RMyc	AGGGATCGCGCTGAGTATAA	TGCCTCTCGCTGGAATTACT
FN/RN2	GCCGGCGGCTGCAGCAAG	TAATTGGCCACAAGGTCTCC
FM2/RM	GGAGACCTTGTGGCCAATTA	CCTGGAGCTGAGGCTGTAAG
FC/RC2	AGCTCGGAGTTGTGAGCACTCTA	TTTTGCCTGCCACAATAACA

Table S2. Size distributions and concentrations of exosomes isolated from malignant *vs* low malignant cancer cells belonging to different types of cancer.

**Metastasis potential**	**Cancer cell line**	**Type of cancer**	**Size (nm)**	**Concentration (particles/ml)**
HIGH	HCC97H	Liver cancer	117	5.97×10^{11}
LOW	HCC97L	Liver cancer	113	5×10^{11}
HIGH	AsPC-1	Pancreatic cancer	102	6.32×10^{11}
LOW	BxPC-3	Pancreatic cancer	107	8×10^{11}
HIGH	HT-29	Colon cancer	129	1.6×10^{11}
LOW	SW480	Colon cancer	134	7×10^{11}
HIGH	DU145	Prostate cancer	106	3.95×10^{11}
LOW	LNCaP	Prostate cancer	119	6.62×10^{11}

Table S3. Source of sera samples, age, gender and stage of cancer patients and healthy controls.

Code	Sample source	Age	Gender	Cancer Type	Stage
BC022S	UCSD Medical Center	37	F	Glioblastoma	IV
BC023S	UCSD Medical Center	47	M	Glioblastoma	IV
BC026S	UCSD Medical Center	52	F	Glioblastoma	IV
BC088VS	UCSD Medical Center	66	M	Glioblastoma	IV
BC138VS	UCSD Medical Center	58	M	Glioblastoma	IV
310	Maine Medical Center	62	F	Breast	Invasive (stage not known)
314	Maine Medical Center	67	M	Breast	Invasive (stage not known)
320	Maine Medical Center	63	M	Colon	IIIB
323	Maine Medical Center	63	F	Breast	IA
326	Maine Medical Center	68	M	Pancreas	IIA
329	Maine Medical Center	72	M	Prostate	IC
331	Maine Medical Center	45	F	Breast	IA
333	Maine Medical Center	72	F	Ovarian	III
334	Maine Medical Center	92	F	Breast	IA
336	Maine Medical Center	36	M	Kidney	I
337	Maine Medical Center	45	F	Cervical	IB
R11-0718	Maine Medical Center	60	F	Ovarian	IA
R11-0902	Maine Medical Center	64	F	Ovarian	Not known
R12-0281	Maine Medical Center	75	F	Ovarian	IIC
R15-0599	Maine Medical Center	73	F	Ovarian	IA
050545	NIH	32	F	Healthy control	
050547	NIH	48	F	Healthy control	
050549	NIH	50	M	Healthy control	
050550	NIH	56	M	Healthy control	
050551	NIH	71	M	Healthy control	
050552	NIH	47	F	Healthy control	
050553	NIH	63	M	Healthy control	

050556	NIH	22	M	Healthy control	
050560	NIH	45	M	Healthy control	
050561	NIH	41	M	Healthy control	
050562	NIH	33	M	Healthy control	
050563	NIH	32	M	Healthy control	
050573	NIH	64	M	Healthy control	
050574	NIH	57	M	Healthy control	
050575	NIH	31	M	Healthy control	
050595	NIH	58	M	Healthy control	
050596	NIH	58	F	Healthy control	
CC	NIH	51	M	Healthy control	
BB	NIH	44	M	Healthy control	
DD	NIH	42	M	Healthy control	
EE	NIH	28	F	Healthy control	
FF	NIH	51	M	Healthy control	
GG	NIH	61	F	Healthy control	
AB	NIH	31	M	Healthy control	
AC	NIH	49	F	Healthy control	
AD	NIH	26	M	Healthy control	
AE	NIH	36	M	Healthy control	
XX	NIH	63	M	Healthy control	
YY	NIH	52	M	Healthy control	
ZZ	NIH	44	M	Healthy control	

Table S4. Size distributions and concentrations of HEK293T exosomes loaded with either CPE-shRNA or CTRL-shRNA.

**Type of ExoHEK**	**Size (nm)**	**Concentration (particles/ml)**
ExoHEK-CPE-shRNA	115.2	7.22×10^{12}
ExoHEK-CTRL-shRNA	116.1	4.37×10^{12}

Supplementary methods

Electron microscopic analysis of exosomes

Exosome pellets, isolated using ExoQuick TC reagent, were fixed in 2.5% glutaraldehyde made in 0.1 M sodium cacodylate buffer (pH 7.4) for 30 minutes at room temperature. The pellets were then rinsed in 0.1M sodium cacodylate buffer, post-fixed in 2% osmium tetroxide made in 0.1M sodium cacodylate buffer, ethanol dehydration series up to 100% ethanol, followed by a Embed-812 resin (Electron Microscopy Sciences) infiltration series up to 100% resin. The epoxy resin was polymerized for 20 h in an oven set at 60°C. Ultra-thin sections (90nm) were prepared on a Leica EM UC7 ultramicrotome. Thin sections were picked up and placed on 200-mesh copper grids (Electron Microscopy Sciences) and post-stained with uranyl acetate and lead citrate. Imaging was accomplished using a JEOL-1400 Transmission Electron Microscope operating at 80kV and images were acquired on an AMT BioSprint 29 camera.
